# Autism Spectrum Disorder Updates – Relevant Information for Early Interventionists to Consider

**DOI:** 10.3389/fpubh.2016.00236

**Published:** 2016-10-28

**Authors:** Paula Allen-Meares, Megan MacDonald, Kristin McGee

**Affiliations:** ^1^University of Illinois at Chicago, Chicago, IL, USA; ^2^College of Public Health and Human Sciences, Oregon State University, Corvallis, OR, USA; ^3^Emerson Waldorf School, Chapel Hill, NC, USA

**Keywords:** ASD, early intervention

## Abstract

Autism spectrum disorder (ASD) is a pervasive developmental disorder characterized by deficits in social communication skills as well as repetitive, restricted or stereotyped behaviors (1). Early interventionists are often found at the forefront of assessment, evaluation, and early intervention services for children with ASD. The role of an early intervention specialist may include assessing developmental history, providing group and individual counseling, working in partnership with families on home, school, and community environments, mobilizing school and community resources, and assisting in the development of positive early intervention strategies (2, 3). The commonality among these roles resides in the importance of providing up-to-date, relevant information to families and children. The purpose of this review is to provide pertinent up-to-date knowledge for early interventionists to help inform practice in working with individuals with ASD, including common behavioral models of intervention.

Public awareness about autism spectrum disorder (henceforth autism or ASD) is growing rapidly as prevalence statistics estimate that 1 in 64 children are diagnosed with autism (4). This means more than a 10-fold increase in prevalence since the 1980s (5). With ASD rates climbing across racial, ethnic, and socio-economic groups, ASD continues to hold the public’s attention as the most common childhood neurodevelopmental disorder (6). Fortunately, up-to-date research is at our fingertips, as the field refines its knowledge about ASD, it is imperative for early interventionists to stay informed about the most current information and best practices, as they relate to early intervention. The purpose of this review is to provide pertinent up-to-date knowledge for clinicians to help inform practice about early intervention and related knowledge.

Autism spectrum disorder is a complex neurodevelopmental disorder that typically presents during toddlerhood (7, 8). The hallmark characteristics of ASD are deficits in social communication skills as well as repetitive, restricted, or stereotypical behavior (1). For example, children with ASD may have difficulty with reciprocal social interaction, joint attention, social initiations, gestures, using body language for non-verbal communication, appropriate facial expressions and eye contact (9, 10). Individuals with ASD may also display repetitive and restricted behaviors. These behaviors are broad but might look like a preoccupation with a specific interest, adherence to a specific routine and repetitive non-functional movements (11). For example, in young children, hand-flapping (stereotypical behavior) or lining up toys (repetitive/restrictive behavior) would characterize such behaviors. In older children with ASD, a preoccupation with a particular television show, animal, or topic of interest would be considered a restrictive behavior. Although we all have our own unique interests, in this context the preoccupation characteristically restricts the child from traditional social interactions, and interrupts daily living routines. For example, it might be difficult to have a conversation without the child bringing the conversation back to their own preoccupied interest or, when a child is hand-flapping he or she may not be focused on learning the task at hand.

The previous iteration of the Diagnostic and Statistical Manual of Mental Disorders, Fourth Edition (12), used pervasive developmental disorder (PDD) as the umbrella term for five unique diagnoses – autistic disorder, Asperger’s disorder, Rett’s disorder, childhood disintegrative disorder (CDD), and PDD – Not Otherwise Specified (PDD-NOS), all of which share deficits in social communication skills as well as a limited range of repetitive or stereotyped activities and interests. The most current edition of the Diagnostic and Statistical Manual of Mental Disorders, Fifth Edition (1) is more focused on one diagnosis of ASD – this restructure from previous iterations of the DSM was focused on commonalities of the unique diagnoses mentioned above (1, 13). Although DSM-IV terminology lingers (e.g., Asperger’s syndrome), it is important to consider the new diagnostic criteria in practice. In other words, similarities in respect to the core characteristics of ASD are routed in early intervention services, yet these characteristics will differ significantly by each individual.

Other comorbid disorders commonly associated with ASD may include, Attention Deficit Hyperactivity Disorder (hyperactivity, short attention span, impulsivity), aggressive behaviors, tantrums, self-injury, chronic sleep problems, atypical eating patterns, over-responsiveness or under-responsiveness to sensory stimulation, and affective difficulties (depression, anxiety) (14). Although these behaviors are common, they are not exclusive to individuals with ASD, nor are they necessary for a diagnosis of ASD.

## Etiology

Researchers and clinicians are working collaboratively and around-the-clock to better understand the etiology of ASD. While there is evidence to suggest genetic underpinnings of ASD, there is no known specific cause of ASD (15–17). While ASD prevalence is higher among boys than girls, it has not been shown to be more prevalent among specific racial, ethnic, or socio-economic groups. Furthermore, while ASD has been primarily considered a neurodevelopmental disorder, there is growing evidence that ASD impacts multiple whole body systems (18). Although an in-depth discussion of the possible etiology of ASD is beyond the scope of this article, we would highly encourage follow-up with recent reviews focused on the following cause theories: genetics (16, 17, 19), environment (18), and obstetric complications and systemizing theory (20).

## Diagnosis

### Importance of Early Diagnosis

Parents of children with ASD tend to notice abnormalities during the child’s first 2 years and many parents notice the first signs of abnormal development before the child’s first birthday (21). The most common concerns are delays in speech and language development (8, 22) followed by abnormal social responsiveness, medical problems, difficulties sleeping and eating, delays reaching milestones, abnormal developmental trajectories, and developmental regression (21, 23–26). The American Academy of Pediatrics (AAP) has recommended universal ASD screening for all young children twice before their second birthday (27). However, in practice the AAP recommendations are not always followed and many children are not diagnosed before age five (28). The importance of early diagnosis is gaining momentum within the ASD community, especially as the understanding of how the disorder presents at younger ages grows (29). Clinically observed behavioral markers of ASD have been recognized well before 24 months of age (30) and more recent findings support reliable diagnosis as young as 12 months of age (7, 31). In essence, early diagnosis leads to earlier eligibility for intervention services, and evidence-based research has clearly indicated that early intervention leads to better prognosis (9, 32–34).

## Screening and Assessment

Although AAP guidelines recommend global screening twice before the second birthday, adherence appears to be lacking (28). The lack of adherence could be due to the subjectivity within screening, screening tools, or specific knowledge about ASD. In part, this may also be due to the plethora of ASD research focused on the latest up-to-date diagnostic and screening mechanisms. As diagnostic criteria change, assessment tools can become difficult to interpret as they are based on outdated information (29, 35). Common screening mechanisms that early interventionist may use to screen ASD include, but are not limited to the modified checklist for autism in toddlers, revised (M-Chat-R™) (36), social responsiveness scale (SRS) (37) and the social communication questionnaire (38).

Evidence-based research indicates the use of multiple sources for the diagnosis of ASD (31, 39). This includes, but is not limited to multiple diagnostic tools, developmental assessment, daily living skills as well as clinical judgment. Currently, the “gold-standard” in autism diagnosis consists of the autism diagnostic observation schedule, second edition (ADOS-2) (7, 10, 40), the autism diagnostic interview-revised (41), and developmental assessments (appropriate for age and level of development at the time of assessment) (29). Additionally, the ADOS-2 and ADI-R have strong interrater reliability as well as strong sensitivity and specificity in the algorithms (31, 42, 43).

A child can be diagnosed with ASD through an educational diagnosis, using criteria from the Individuals with Disabilities Education Act (IDEA), where the purpose of diagnosis is to indicate if the child qualifies for special education services (44). One limitation to an educational diagnosis is that children have already entered school; therefore, it is “too late” to take advantage of early intervention services. In addition, educational diagnoses have been less aligned with DSM-V criteria; thus, it is possible that some students are missed, within an educational diagnosis. Clinicians and early intervention specialists on the forefront of meeting families and recognizing ASD characteristics in children need to thoughtfully consider best practice in diagnosis and signs that may be present at an early age.

## Treatment and Intervention

The hallmark characteristics of ASD are deficits in social communication skills as well as repetitive and restricted behaviors (1, 11). For the most part interventions are driven by these hallmark characteristics. For example, interventions have focused on improving social communication skills, such as language skills, play, and reciprocal communication. The necessity of early intervention has been clearly indicated as a priority in autism research (34, 45). Findings indicate that children who enter early intervention have a better prognosis (46, 47). In 2010, the first randomized clinical control trial of an early intervention for children with autism was published (46). Young pre-school aged children who received this early intervention had improved IQ, language, adaptive behavior, and a better diagnostic prognosis. Other early intervention studies for young children with autism have also had promising results, including but not limited to, better joint attention skills, daily living skills, and ultimately better social and communicative behaviors (9, 33, 34, 48). Amidst this paucity of research promising findings indicate that early intervention has far-reaching positive effects, especially when children enter intervention at a young age. Research about early intervention for children with autism is ongoing, but best practice recommendations suggest: early entry, intensive instruction all day (representative of a school-day) 5-days per week, year-round, and inclusive settings (45, 49, 50).

Early intervention is a priority of ASD research and consensus among professionals suggests any type of intervention is better than no intervention (34). Current research is testing how intervention types compare to each other to better understand if one modality is better than another. This section reviews widely used intervention strategies – however, it is important to note that many of these intervention methods are continually being assessed for content.

### Treatment Modalities

Treatment modalities for ASD can be divided into three broad categories: psycho-educational or behavioral models, psychopharmacological models, and alternative and complementary models (51, 52). This article will discuss some popular behavioral models.

## Behavioral Intervention Models

One of the earliest documented and most widely cited early interventions for young children with ASD is applied behavioral analysis (commonly known as ABA) (53). Positive results from this intensive 40-h per week behavioral intervention include improvements in intellectual and educational functioning (53). A similar, yet unique early intervention is discrete-trial training (DTT). Proponents of DTT view ASD as a multitude of unique behaviors and reject the idea of one central deficit that can be found in all individuals with ASD.

Discrete-trial training is based on operant-conditioning behavioral models – with reinforcement control as the basis for behavior change. In practice, clinicians and educators use reinforcement, backward chaining, shaping, prompting, and prompt fading to implement DTT. DTT relies on intensive discrete-trial sessions that consist of four parts: (1) the trainer’s presentation of stimuli, (2) the child’s response, (3) the consequence, and (4) a short pause prior to the next stimuli (54). There are two phases, phase one engages 40 h per week of one-on-one DTT, administered by trained DTT professionals and the children’s parents over a 1- to 2-year period. Phase two focuses on expressive and receptive language skills, abstract play, and social play and uses both DTT and generalization to playgroup and/or supported preschool experiences (55). The behavioral principles of DTT can be successfully applied to children with ASD and the goal is that when placed in environments utilizing DTT, children will emulate typical learning patterns (56). Criticisms of DTT include a loose relationship between the method rationale and the diagnosis of ASD, a narrow approach to language development, the need for “prompt” dependence, and the high cost of the program (57). A controlled study has been conducted and although initial results suggested success, follow-up studies indicated that the learned skills did not improve at a level consistent with peer developmental trajectories (58). Other methodological issues included the lack of random assignment, participant-sampling bias toward higher functioning children with ASD, and assessors who were not blind to study participants (57). In addition, other studies have failed to show similar results to the original controlled study. Yet, DTT is commonly used in practice.

The Pivotal Response Model has no age restriction and has shown effectiveness for increasing positive behavior and decreasing negative behaviors in children of all ages. The literature suggests that the Pivotal Response Model is the most useful in young children, as an early intervention (59). This model is based on the principle that intervention in a few core (or pivotal) areas will increase skills in all areas (even those not directly targeted) and decrease problem behaviors. The pivotal areas include motivation, multiple cues, self-management, and the initiation of social interaction (59). Outcome studies have reported improvements in speech and language, social skills, and generalizing learned skills beyond the treatment setting (60, 61).

Treatment and Education of Autistic and related Communication-Handicapped Children (TEACCH) was originally developed for children, but is now used as an intervention at all ages. The overarching goal of TEACCH seeks to work toward participant independence (57). Key principles from the TEACCH model include, careful ongoing assessment, using the strength of the participant as a building block, the use of environment embedded within the behavioral framework and the involvement of parents.

The TEACCH intervention includes diagnosis, parent training, education, social and leisure skills development, communication, vocational training, and supported employment (57). Behavioral strategies include the use of schedules, a visual independent work system, and clearly organized instructional materials to create a structured and predictable learning environment. If children’s progress becomes hindered, then the environment is often modified to accommodate the identified issue (62). TEACCH has successfully improved self-help skills, social skills, and communication, enhancing quality of life and reduced inappropriate behavior (63). When children partaking in TEACCH were compared with matched peers participating in DTT, the TEACCH children had outcomes three to four times greater than the control group (DTT) on all measures (64). The results of this study should be interpreted with caution as there was no random assignment; therefore, it is hard to draw concrete conclusions between the success of the two programs. Although TEACCH is a widely used intervention strategy, no large, well-controlled study has been conducted to assess its effectiveness (57, 65).

The use of Social Stories is a common behavioral intervention in which short, simple stories written from the perspective of the child are used to deliver instruction on appropriate social behavior. These stories are carefully designed to be within the comprehension level of the child and can be used with younger and older children accordingly (66). Although Social Stories are widely accepted due to the connection to prevailing theories of autism, evidence-based research is needed. Case studies as well as other experimental design studies that have been conducted but indicate mixed results. Workshops for parents, teachers, and assistants have been successful toward implementing the use of social stories in the participants’ respective environment (67). Other case studies about Social Stories have also displayed mixed results.

The use of visual supports is particularly popular and useful in working with individuals with autism. One such approach is the Picture Exchange Communication System (PECS), which aims to build language skills and teach communication response and initiation (57). Pictures are used to make requests and to form simple sentences. It is a low-cost intervention that does not depend on eye contact or the training of multiple partners, is compatible with TEACCH and the Lovaas method, and works well with pre-verbal or non-verbal children (57).

Many behavioral early interventions consist of specific techniques and require dedicated time by an interventionist or caregiver, often consisting of up-to 40 h per week of practice. More general best practice recommendations for early intervention include building skills into daily routines, the use of natural environments, and about 25 h per week of direct skill practice (68). It should be noted that successful early interventions have been indicated with as little as 1 h per week (69), while more standard practice is about 40 h per week (56).

## A Special Focus on ASD in Educational Settings

### Background on Individuals with Disabilities Education Act

The IDEA passed in 1990 and its successor, the Individuals with Disabilities Education Improvement Act (IDEIA, still referred to as IDEA), was reauthorized in 2004. IDEA protects the rights of children with disabilities and the parents of the children, guarantees that children with disabilities have an appropriate public education adhering to their unique needs, at no cost (Building the Legacy: IDEA 2004, 2010).

Individuals with Disabilities Education Act has its roots in the Education for All Handicapped Children’s Act of 1975 (PL 94–148) that provided federal funding to states for free, appropriate public education to children with disabilities. Amendments throughout the 1980s and 1990s added provisions for vocational training and transition services, services, and programs for children birth to age 3 years, transition planning for teenagers, and mandates that schools report children’s progress to parents (Building the Legacy: IDEA 2004, 2010) (70). The 2004 reauthorization of the IDEA as well as President George W. Bush’s Commission on Excellence in Special Education made attempts to revamp inefficiencies in the special education system.

Individualized Education Plans (IEP) are the foundation of special education services provided to children with disabilities in the public school system. Inclusion requirements for an IEP are mandated at the federal and state level; furthermore, it is mandated that students are educated in the least restricted environment (e.g., students should be placed in general education settings, when the necessary supports are in place and if this meets the needs of the student). Federal requirements consist of a statement of the child’s present levels of academic achievements and functional performance, measurable annual goals, a description of benchmarks, a description of how the child’s progress toward meeting the annual goals will be measured, and a description of when periodic reports on the progress the child is making toward meeting annual goals will be provided (concurrent with issuance of report cards) (Building the Legacy: IDEA 2004, 2010). When a child turns 16 years of age, the IEP must include a statement of appropriate measurable postsecondary goals related to training, education, employment, and/or independent living skills and a statement of transition services needed to reach those goals. IEPs should allow the child to make progress in the general education curriculum as well as other educational curriculum as needed on an individual basis (Building the Legacy: IDEA 2004, 2010).

### ASD, IDEA, and IEPs

Designing IEPs, specific to individuals with ASD, the objectives, target behaviors, and levels of supports should all be clearly defined, specific, and developmentally appropriate (14). Specific IEP goals should be individualized, but reflective of common characteristics of autism, including communication goals related to requesting, labeling, identifying, following directions, making conversation, using spontaneous and generalized communication skills, greeting, and using and understanding non-verbal communication. Social interaction goals and objectives for an IEP could include joint attention and early social engagement skills, social play, pretend play, consideration of the perspective and feelings of others, friendship, social skills, and problem-solving (14). Lastly, goals and objectives related to restricted and repetitive behaviors could include managing and reducing or eliminating stereotypic behaviors, understanding and demonstrating flexibility, and managing and reducing obsessive thoughts and compulsive behaviors. Other consideration’s within IEP goals might reflect behaviors related to emotional self-regulation (mood, anxiety) and behavior management (self-injury, aggression, anger management, staying on-task), academic skills (pre-academic skills, critical thinking, working/being in a group), adaptive skills (feeding, sleeping, dressing, toileting, self-care and self-management, functional independent play, daily living skills, participation in family and community, leisure skills, vocational skills, transition to adulthood) (14).

## Implications for Clinicians

Interventions targeted at social communicative behaviors are necessary for children of all ages diagnosed with autism. Behavioral, personal, and environmental circumstances are all taken into consideration in developing intervention models (69). Early intervention has been particularly targeted due to the positive prognosis indicated through evidence-based research (9, 33, 34, 46). There is widespread agreement on the necessity of early intervention but there is less consistent agreement on specific content (34). As little as 6 h of parent training (didactic group sessions for parents) has been shown to have positive effects on the child’s social communicative behaviors (26, 69). On the contrary, more than 40 h of intensive intervention for most waking hours of the child’s day has been advocated (53). Standardized diagnostic measures identify children with autism at an early age have been successful (7, 31), but providing immediate and effective resources to help families cope with the new diagnosis is lacking (49). Parent-implemented interventions aimed at promoting socialization and communication have had successful results, but more work is needed in order to provide clear instructions and design user-friendly manuals with relevant, easy-to-understand parental resources for families of children with ASD (71). Even though early intervention for some of the youngest children with ASD appear on the forefront of research initiatives, more work is needed to establish effective, meaningful, and parent-friendly intervention techniques (71). Clinicians play a meaningful role in addressing the necessary steps toward active participation in early intervention. Starting with assessment, and finishing with the right intervention “fit” for children and the family.

It is of the utmost importance that clinicians disseminate current research (35). In short, clinicians are most likely to assist children and families in connecting the dots. Clinicians are often the first person, aside from the family, to acknowledge ASD characteristics. Becoming familiar with up-to-date research and better understanding ASD resources within the community will help establish a seamless transition from the clinic to behavioral interventions and further assist families in learning more about how to provide the necessary supports to their child with ASD.

## Author Contributions

This manuscript was conceptualized and developed by PA-M; MM and KM assisted with manuscript writing.

## Conflict of Interest Statement

The authors declare that the research was conducted in the absence of any commercial or financial relationships that could be construed as a potential conflict of interest. The reviewer MS, AB, and handling Editor declared their shared affiliation, and the handling editor states that the process nevertheless met the standards of a fair and objective review.
